# Ophiopogonin A Alleviates Hemorrhagic Shock-Induced Renal Injury via Induction of Nrf2 Expression

**DOI:** 10.3389/fphys.2020.619740

**Published:** 2021-02-01

**Authors:** Xiaoming Sheng, Yang Yang, JiaJia Liu, Junbo Yu, Qingsong Guo, Wei Guan, Fan Liu

**Affiliations:** ^1^Department of Trauma Center, Affiliated Hospital of Nantong University, Nantong, China; ^2^School of Pharmacy, Nantong University, Nantong, China; ^3^Department of Orthopaedics, Affiliated Hospital of Nantong University, Nantong, China

**Keywords:** ophiopogonin A, hemorrhagic shock-induced renal injury, Nrf2, p-ERK/ERK, renal injury

## Abstract

Ophiopogonin, including Ophiopogonin A, B, C, D, is an effective active component of traditional Chinese medicine *Ophiopogon japonicus* which has a wide range of pharmacological effects such as protecting myocardial ischemia, resisting myocardial infarction, immune regulation, lowering blood glucose, and anti-tumor. However, the functions of ophiopogonin A on hemorrhagic shock (HS)-induced renal injury remain unclear. First, this study constructed an HS rat model and hypoxia HK-2 cell model to assess the effects of ophiopogonin A *in vivo* and *in vitro*. *In vivo*, HE and TUNEL staining show that ophiopogonin A dose-dependently inhibits HS-induced tissue damage and apoptosis. Moreover, ophiopogonin A dose-dependently downregulates the levels of blood urea nitrogen (BUN), creatinine (Cr), KIM-1, NGAL, iNOS, TNF-α, IL-1β, and IL-6 in HS rats kidney tissues, and decreases the number of MPO-positive cells. *In vitro*, we get similar results that ophiopogonin A dose-dependently improves hypoxia-induced HK-2 cell apoptosis and damage. In addition, ophiopogonin A dose-dependently increases the expression of NF E2-related factor 2 (Nrf2), while knockdown of Nrf2 reverses the functions of ophiopogonin A *in vivo* and *in vitro*. Furthermore, ophiopogonin A dose-dependently promotes the phosphorylation of ERK in HS kidney tissues and hypoxia-treated HK-2 cells, suggesting that ophiopogonin A functions via the p-ERK/ERK signaling pathway.

## Introduction

Hemorrhagic shock (HS) is one of a number of critical clinical situations that can be caused by trauma or major surgery. A decrease in blood perfusion in multiple tissues and organs across the whole body is caused by HS, which leads to complications and a high mortality rate (Mitra et al., 2014; Hamada et al., 2020). It has been reported that severe hemorrhage creates sympathetic nerve excitement, which then leads to a significant reduction in renal blood perfusion, causing renal ischemia and hypoxia, resulting in acute kidney injury (AKI) and accelerating the death of the patient (Wu et al., 2020). AKI is a common clinical disease, especially in critically ill patients who have undergone major surgery, developed sepsis, experienced trauma, cardiogenic shock, hypovolemia, or used nephrotoxic drugs. Although the technology for the treatment of kidney disease continues to advance, the fatality rate and disability rate of AKI remains high. According to a study published by *The Lancet* in 2017, approximately 4.61 million people die of trauma each year worldwide, accounting for 8.43% of global deaths (GBD 2016 Causes of Death Collaborators, 2017), and 40% of trauma patients die from HS (Cothren et al., 2007). It is therefore essential to develop new treatment strategies for renal injuries caused by HS.

The mechanism of HS-induced kidney injury is complex. An increasing number of reports demonstrate that oxidative stress and inflammatory response, microcirculation dysfunction play critical roles in the progression of HS-induced kidney injury (Harrois et al., 2017). It has been reported that the release of proinflammatory cytokines, including TNF-α, IL-6, and IL-1β, as well as iNOS caused by HS, participate in the development of kidney injury (Chen et al., 2015, 2017; Rabb et al., 2016). Therefore, inhibition of the inflammation response and iNOS are the key to treating the kidney injuries induced by HS. In addition, Nrf2 is an important transcription factor against oxidative stress in the body. Nrf2 deficiency or activation disorder will lead to the intolerance or decline of tolerance of cells to oxidative stress, increasing the negative effect of oxidative stress, and leading to cell dysfunction, apoptosis, and even death. To date, many experiments and studies have confirmed the protective role played by Nrf2 in a series of diseases with high oxidative stress, such as acute respiratory distress syndrome, emphysema, pulmonary fibrosis, cirrhosis, and colitis (Rangasamy et al., 2004; Reddy et al., 2009; Tang et al., 2016; Yang et al., 2016). Recent studies report on the protective effects of Nrf2 in local cerebral ischemia and renal ischemia. Nrf2 protects brain cells and renal cells by regulating the increase of glutathione synthesis and scavenging oxygen free radicals in ischemic brain tissue and renal tissue (Liu et al., 2009; Righy et al., 2016). However, the role of Nrf2 in renal injury caused by hemorrhagic shock and its regulatory mechanism are still unknown. This study aimed to investigate the protective effect of Nrf2 on renal injury caused by hemorrhagic shock and its mechanism, to provide a new target and theoretical basis for the clinical treatment of renal injury caused by hemorrhagic shock.

A growing body of research demonstrates that ingredients used in Traditional Chinese Medicine including alkaloid, terpenoid, polysaccharide, and acid compound, are effective and can play a key role in treating various diseases (Hao et al., 2017; Li and Le, 2019). For instance, Artemisinin, derived from sweet wormwood, has effective antimalarial pharmacotherapy and anti-tumor effects (Tu, 2016; Wong et al., 2017). Paclitaxel, a complex diterpenoid compound extracted from the bark of *Taxus brevifolia*, has a spectrum and anti-cancer activity, especially for uterine cancer, ovarian cancer, and breast cancer (Fader et al., 2018; Xiang et al., 2019). Ophiopogonin is an active ingredient extracted from Ophiopogonis Radix, including Diosgenin (Ophiopogonin B’, C’, and D’) and Ruscogenin (A, B, C, and D), that plays a role in delaying aging, improving learning and memory disorders, anti-cardio-cerebrovascular diseases, anti-tumor, anti-inflammatory, and immune regulation (Wang et al., 2018; Lu Z. et al., 2020; Zhu et al., 2020). In the mouse model of middle cerebral artery occlusion (MCAO), Guan et al. (2013) found that Ruscogenin protects against ischemic brain injury by inhibiting the NF-κB mediated inflammatory pathway. In addition, Ruscogenin reduces the effect of LPS-induced lung injury by downregulating the expression of TF, NF-κB-p65, and iNOS (Sun et al., 2012). All these findings suggest that Ophiopogonin might have therapeutic effects on tissue injury caused by physicochemical agents. There are few studies on Ophiopogon A and kidney damage caused by hemorrhagic shock. Our study aimed to test the protective effect of ophiopogonin A on HS-induced kidney injury and explore whether its mechanism was related to the Nrf2/ERK signaling pathway.

## Materials and Methods

### The HS Rat Model

Male Sprague-Dawley rats (6–8 weeks, 230–260 g) were purchased from the Experimental Animal Center of Shanghai Jiao Tong University (Shanghai, China). Animal protocols were approved by the Institutional Animal Care and Use Committee (IACUC). An HS model was established in the rats according to previous research (Du et al., 2013). In brief, after anesthetizing by intraperitoneal injection of 10% chloral hydrate, PE-50 tubes were inserted into the bilateral femoral artery and the right femoral vein, and the left femoral artery was used to draw blood for the shock model. Mean arterial pressure (MAP) was monitored and the MAP and controlled at (40–50) mmHg for 90 min. The right femoral vein was used for resuscitation after shock. The extracted blood and the same amount of normal saline were reinfused. Ophiopogonin A was given by intraperitoneal injection at the beginning of the resuscitation, Nrf2-related lentivirus (10^9^ PFU/ml, 200 μl) system plasmids were constructed to collect lentiviruses and injected into rats via the tail vein. After 4-h resuscitation, the rats were killed and samples were collected for the following experiments.

### Hypoxia Model *in vitro*

HK-2 cell was obtained from Cobioer (Nanjing, China). The cells were cultured with DMEM containing 10% fetal bovine serum and 1% penicillin/streptomycin at 37°C, 5% CO_2_ incubator. As outlined in a previous study (Liu et al., 2017), a hypoxia glove box was used to establish the hypoxia environment of HK-2 cells. Nitrogen gas was infused into the hypoxia glove box to maintain the internal environment at 37°C, 5% CO_2_, and 0.3% O_2_.

### Hematoxylin and Eosin (HE) Staining and TUNEL Assay

The rat’s kidney was collected and fixed in 10% formalin. Next, the paraffin sections were dewaxed and hydrated; and then slices were incubated in HE solution for 5–20 min. Finally, the kidney slices were observed in a light microscope (Olympus, Tokyo, Japan), and kidney damage was assessed according to previous research. The apoptosis was detected by TUNEL assay based on protocols (Beyotime, Shanghai, China).

### CCK-8 Assay

A Cell Counting Kit 8 (CCK-8; Apexbio, Houston, TX, United States) was used to detect HK-2 cell growth. Briefly, the cells were incubated in a 96-well assay for 24, 48, or 72 h. Then, the cells were treated with the CCK-8 solution. Next, the plate was incubated at 37°C for 3 h. The optical density (OD) value was detected at 450 nm by ultraviolet spectrophotometer (Thermo Fisher Scientific, Inc.).

### qRT-PCR Analysis

RNA was extracted from cell and tissue samples using TRIzol reagents (Invitrogen, Shanghai, China). The PrimeScript^TM^ II 1st Strand cDNA Synthesis Kit (Takara, Otsu, Japan) was used to induced cDNA. Quantitative real-time quantitative PCR (qPCR) was performed using SYBR^®^ green main mixture in a Real-time fluorescence quantitative PCR instrument (Bio-Rad, CA, United States). The primers for RT-qPCR are shown in Table 1.

**TABLE 1:** 

Primer sequences used for qRT-PCR in Rats.		

Genes		Primer sequences (5′-3′)
KIM-1	Forward	ACTCCTGCAGACTGGAATGG
	Reverse	ACTCCTGCAGACTGGAATGG
NGAL	Forward	GATGAACTGAAGGAGCGATTC
	Reverse	TCGGTGGGAACAGAGAAAAC
Nrf2	Forward	TCTGACTCCGGCATTTCACT
	Reverse	GGCACTGTCTAGCTCTTCCA
β-actin	Forward	TCAGGTCATCACTATCGGCAAT
	Reverse	AAAGAAAGGGTGTAAAACGCA

**Primer sequences used for qRT-PCR in HK-2 cells.**		

**Genes**		**Primer sequences (5′-3′)**

KIM-1	Forward	TGGCAGATTCTGTAGCTGGTT
	Reverse	AGAGAACATGAGCCTCTATTCCA
NGAL	Forward	CCACCTCAGACCTGATCCCA
	Reverse	CCCCTGGAATTGGTTGTCCTG
Nrf2	Forward	TCAGCGACGGAAAGAGTATGA
	Reverse	CCACTGGTTTCTGACTGGATGT
β-actin	Forward	CTCCATCCTGGCCTCGCTGT
	Reverse	GCTGTCACCTTCACCGTTCC

### Western Blot Analysis

Total protein was extracted by RIPA lysis buffer. After protein extraction, a BCA protein assay kit (Thermo Fisher Scientific, MA, United States) was used to detect protein concentration. Protein samples were added to the band and fractionated by 10% Bis-Tris gel, then transferred to PVDF membranes. After being blocked by 5% non-fat milk, the membranes were incubated with primary antibody, respectively, at 4°C overnight. Then the membranes were incubated for 2 h with HRP-conjugated secondary antibody (1:5000 dilution) at room temperature. ECL Western blotting substrate (Vazyme, Nanjing, China) was applied to detect PVDF membranes. Image J software was then used to quantitatively detect protein bands.

### Detection of BUN, Cr, and Inflammatory Factors

The content of BUN and Cr in rat serum samples was measured by automatic biochemical analyzer (Icubio, Shenzhen, China). The content of TNF-α, IL-1β, and IL-6 was tested by ELISA assay (Mlbio, Shanghai, China).

### Immunohistochemistry

Myeloperoxidase-positive cells were measured by immunohistochemistry according to a previous study (Sordi et al., 2019).

### Statistical Analysis

Results were collected from three independent experiments. All the results were presented as means ± SD. Results were analyzed with one-way ANOVA and *t*-test using GraphPad Prism 7.0 (GraphPad Inc., San Diego, CA, United States). *P* < 0.05 was considered statistically significant.

## Results

### Ophiopogonin A Alleviates Renal Injury in HS Rats’ Model

**FIGURE 1:**
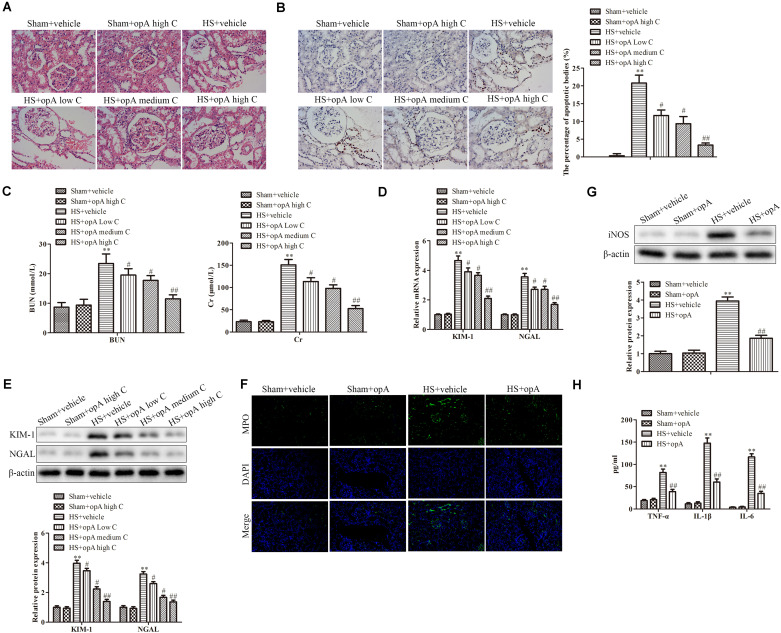
Ophiopogonin A alleviates renal injury in the HS rat model. **(A)** Representative kidney HE staining of sham + vehicle (*n* = 6), sham + ophiopogonin A high c (*n* = 6), HS + vehicle (*n* = 6), HS + ophiopogonin A low c (*n* = 6), HS + ophiopogonin A medium c (*n* = 6), and l HS + ophiopogonin A high c (*n* = 6) groups. **(B)** Representative kidney TUNEL staining. **(C)** The changes of serum creatinine and BUN in rats. **(D)** Relative mRNA expression of KIM-1 and NGAL in the kidney. **(E)** Relative protein expression of KIM-1 and NGAL in the kidney. **(F)** MPO-positive cells recruitment in kidney detected by immunohistochemistry. **(G)** The expression of iNOS in rats. **(H)** The amounts of TNF-α, IL-1β, and IL-6 determined by ELISA assay. Data were presented as mean ± SD. ^∗∗^*P* < 0.01, ^#^*P* < 0.05, ^##^*P* < 0.01 compared with the corresponding control group.

To investigate the functions of ophiopogonin A on renal injury induced by HS, kidney histological changes were detected. As shown in Figure 1A, high concentrations of ophiopogonin A did not affect normal rat kidney tissue. Compared with the normal rat kidney group there was pathological damage, including cell necrosis, loss of brush border, cast formation, and tubule dilation in the HS group, while ophiopogonin A partly improved HS-induced tissue damage, especially high concentration of ophiopogonin A. Furthermore, TUNEL staining showed that ophiopogonin A markedly inhibited HS-induced cell apoptosis in the rat kidneys, and the most effective results were observed in high doses of ophiopogonin A (Figure 1B). Blood urea nitrogen (BUN) and creatinine (Cr) are indicators of renal insufficiency (Vaidya et al., 2010). To assess the effects of ophiopogonin A on renal function, the levels of BUN and Cr were measured. There was no significant difference in the levels of BUN and Cr between sham + vehicle and Sham + ophiopogonin A high C group (Figure 1C). Compared to control groups, the levels of serum BUN and Cr were increased in the HS group, which were pronouncedly reversed by ophiopogonin A, especially in high concentrations (Figure 1C). Moreover, neutrophil gelatinase-associated lipocalin (NGAL) and kidney injury molecule (KIM-1) acted as a diagnostic marker to evaluate the severity and prognosis of kidney injury (Hoste et al., 2020). Our study found that the mRNA and protein expression of KIM-1 and NEAL were upregulated in the HS group compared with the sham group, while ophiopogonin A at dose-dependence reversed HS-induced these phenomena (Figures 1D,E). In addition, Myeloperoxidase (MPO) was a specific marker of local neutrophil accumulation, which indicated the extent of kidney damage. In this study, the kidney of HS-treated with vehicle exhibited an upregulation in the expression of MPO compared with sham rats, however, ophiopogonin A could recover HS-induced MPO change (Figure 1F). On the other hand, the expression levels of iNOS, TNF-α, IL-1β, and IL-6 were significantly upregulated in the HS + vehicle group compared with the sham + vehicle group, while ophiopogonin A inhibited HS-induced iNOS, TNF-α, IL-1β, and IL-6 expression (Figures 1G,H). The above data indicates that a high concentration of ophiopogonin A significantly alleviated HS-induced renal injury in rats.

### Ophiopogonin A Decelerates Hypoxia-Induced Apoptosis and Damage in HK-2 Cells

**FIGURE 2:**
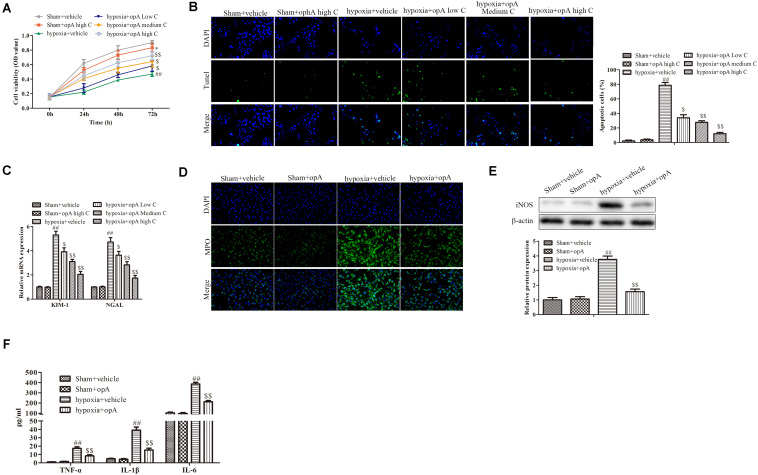
Ophiopogonin A decelerates hypoxia-induced apoptosis and damage in HK-2 cells. **(A)** Cell viability measured by CCK-8 assay. **(B)** Cell apoptosis detected by TUNEL staining in HK-2 cells. **(C)** The expression of KIM-1 and NGAL. **(D)** MPO-positive cells recruitment detected by immunohistochemistry. **(E)** The expression of iNOS in HK-2 cells. **(F)** The amounts of TNF-α, IL-1β, and IL-6 determined by ELISA assay. Data were presented as mean ± SD. ^∗^*P* < 0.05, ^##^*P* < 0.01, ^$^*P* < 0.05, ^$$^*P* < 0.01 compared with the corresponding control group.

To investigate the functions of ophiopogonin A *in vitro*, HK-2 cells were used to establish the hypoxia model. CCK-8 assay demonstrated that a high concentration of ophiopogonin A had no significant effect on cell viability in the Sham + vehicle group, while hypoxia inhibited cell viability compared to sham with the vehicle group (Figure 2A). Ophiopogonin A could reverse the inhibition effects of hypoxia on cell viability (Figure 2A). In addition, ophiopogonin A also inhibited hypoxia-induced HK-2 cell apoptosis in a dose-dependent manner through TUNEL staining (Figure 2B). Consistent with the experimental results in rats, ophiopogonin A inhibited hypoxia-induced KIM-1, NGAL, MPO, iNOS, TNF-α, IL-1β, and IL-6 expression in HK-2 cells, especially in high concentrations (Figures 2C–F). These results demonstrate that ophiopogonin A decelerated hypoxia-induced apoptosis and damage *in vitro*.

### Ophiopogonin A Promotes the Expression of Nrf2 *in vivo* and *in vitro*

**FIGURE 3:**
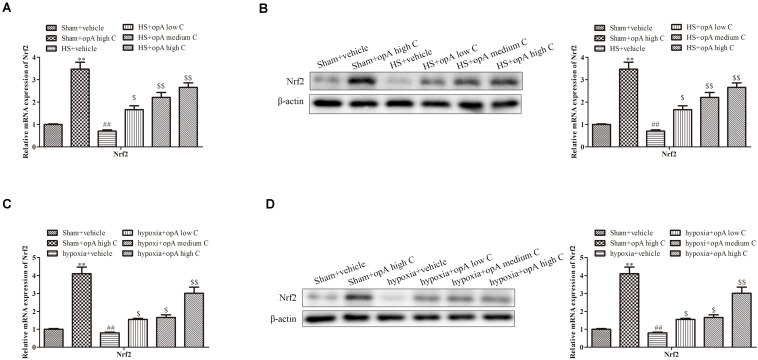
Ophiopogonin A promotes the expression of Nrf2 *in vivo* and *in vitro*. **(A)** Relative mRNA expression of Nrf2 in the kidney. **(B)** Relative protein expression of Nrf2 in the kidney. **(C)** Relative mRNA expression of Nrf2 in HK-2 cell. **(D)** Relative protein expression of Nrf2 in HK-2 cell. ^∗∗^*P* < 0.01, ^##^*P* < 0.01, ^$^*P* < 0.05, ^$$^*P* < 0.01 compared with the corresponding control group.

Studies showed that Nrf2 played critical roles in kidney injury. We thus hypothesized that ophiopogonin A alleviates kidney injury through regulating Nrf2. To explore the effects of ophiopogonin A on Nrf2 expression, *in vitro* and *in vivo* experiments were measured. qRT-PCR and western blot analyses revealed that compared to the control group, ophiopogonin A promoted the expression of Nrf2 in both the sham group and HS group, especially in high concentrations (Figures 3A,B). Consistently, similar results were obtained on HK-2 cells, which ophiopogonin A promoted the expression of Nrf2 (Figures 3C,D).

### Knockdown of Nrf2 Partly Blocks the Functions of Ophiopogonin A *in vivo*

**FIGURE 4:**
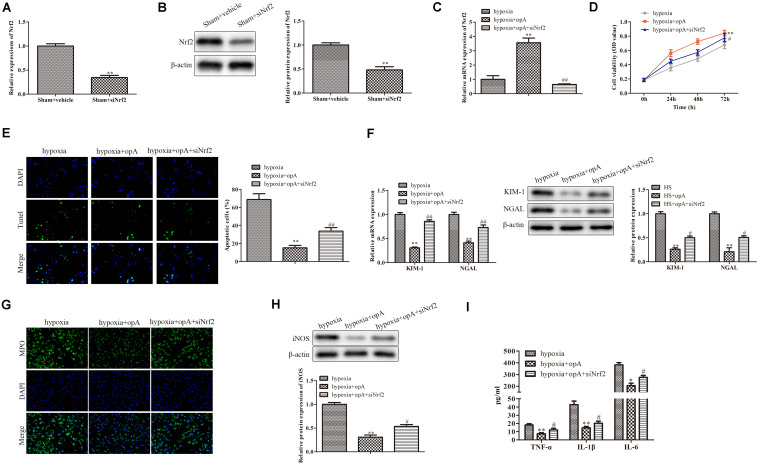
Knockdown of Nrf2 reverses the functions of ophiopogonin A *in vivo*. **(A)** Relative mRNA expression of Nrf2 in sham and sham + siNrf2 group *in vivo*. **(B)** Relative protein expression of Nrf2 in sham and sham + siNrf2 group *in vivo.* **(C)** Relative mRNA expression of Nrf2 in HS, HS + ophiopogonin A, and HS + ophiopogonin A + siNrf2 group. **(D)** Relative mRNA expression of Nrf2. **(E)** Relative protein expression of Nrf2. **(F)** The changes in serum creatinine and BUN in rats. **(G)** MPO-positive cells recruitment in kidney detected by immunohistochemistry. **(H)** The expression of iNOS in rats. **(I)** The amounts of TNF-α, IL-1β, and IL-6 determined by ELISA assay. Data were presented as mean ± SD. ^∗∗^*P* < 0.01, ^∗^*P* < 0.05, ^#^*P* < 0.05, ^##^*P* < 0.01 compared with the corresponding control group.

To further investigate the roles of Nrf2 on kidney injury, ophiopogonin A and siNrf2 were used to treat HS rats. First, we detected the transfection efficiency of siNrf2 by qRT-PCR and western blot analysis, the results showed that siNrf2 transfection markedly inhibited the expression of Nrf2 (Figures 4A,B). As shown in Figure 4C, knockdown of Nrf2 decreased ophiopogonin A-induced Nrf2 expression in HS rats. qRT-PCR and western blot analyses demonstrated that the expression of KIM-1 and NGAL was increased in the HS + ophiopogonin A + siNrf2 group, compared with HS + ophiopogonin A group (Figures 4D,E). Besides, knockdown of Nrf2 significantly attenuated ophiopogonin A-induced decline of serum Cr and BUN levels in HS rats (Figure 4F). Likewise, knockdown of Nrf2 increased MPO-positive cells in HS-rats with ophiopogonin A (Figure 4G). The expression of iNOS, TNF-α, IL-1β, and IL-6 was largely upregulated in the HS + ophiopogonin A + siNrf2 group compared with HS + ophiopogonin A group (Figures 4H,I). All data illustrated that Nrf2 silencing recovered the effects of ophiopogonin A in HS rats.

### Silencing of Nrf2 Partly Blocks the Functions of Ophiopogonin A *in vitro*

**FIGURE 5:**
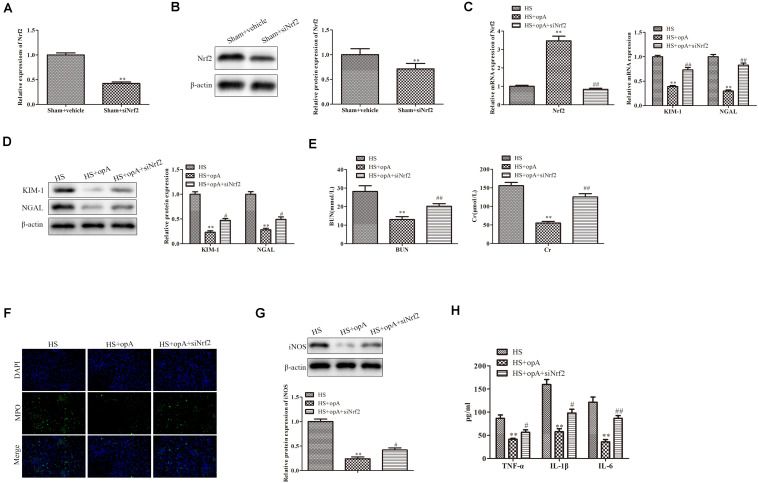
Silencing of Nrf2 reverses the functions of ophiopogonin A *in vitro*. **(A)** Relative mRNA expression of Nrf2 in sham and sham + siNrf2 group *in vitro*. **(B)** Relative protein expression of Nrf2 in sham and sham + siNrf2 group *in vitro.* **(C)** Relative mRNA expression of Nrf2 in hypoxia, hypoxia + ophiopogonin A and hypoxia + ophiopogonin A + siNrf2. **(D)** Cell viability detected by CCK-8 assay in HK-2 cell. **(E)** Cell apoptosis assessed by TUNEL staining. **(F)** MPO-positive cells recruitment detected by immunohistochemistry. **(G)** The expression of iNOS in HK-2 cells. **(H)** The amounts of TNF-α, IL-1β, and IL-6 determined by ELISA assay. Data were presented as mean ± SD. ^∗∗^*P* < 0.01, ^#^*P* < 0.05, ^##^*P* < 0.01 compared with the corresponding control group.

We then further explored the roles of Nrf2 knockdown *in vitro*. First, the knockdown efficiency of Nrf2 was detected by western blot and qRT-PCR analysis. The results revealed that the knockdown of Nrf2 significantly downregulated the expression of Nrf2 (Figures 5A,B). We also found that siNrf2 reversed hypoxia-induced Nrf2 upregulation in HK-2 cells (Figure 5C). CCK-8 assay showed that cell viability was inhibited by Nrf2 knockdown in the hypoxia + ophiopogonin A group (Figure 5D). The silencing of Nrf2 reversed ophiopogonin A-induced cell apoptosis in hypoxia-treated HK-2 cells by TUNEL staining (Figure 5E). The decreased expression of MPO, iNOS, TNF-α, IL-1β, and IL-6 in the hypoxia + ophiopogonin A group were also blocked by Nrf2 knockdown (Figures 5F–H). These results verified that the knockdown of Nrf2 recovered the functions of ophiopogonin A in hypoxia-treated HK-2 cells.

### Ophiopogonin A Regulates Nrf2 Expression by Activation ERK Signaling Pathway

**FIGURE 6:**
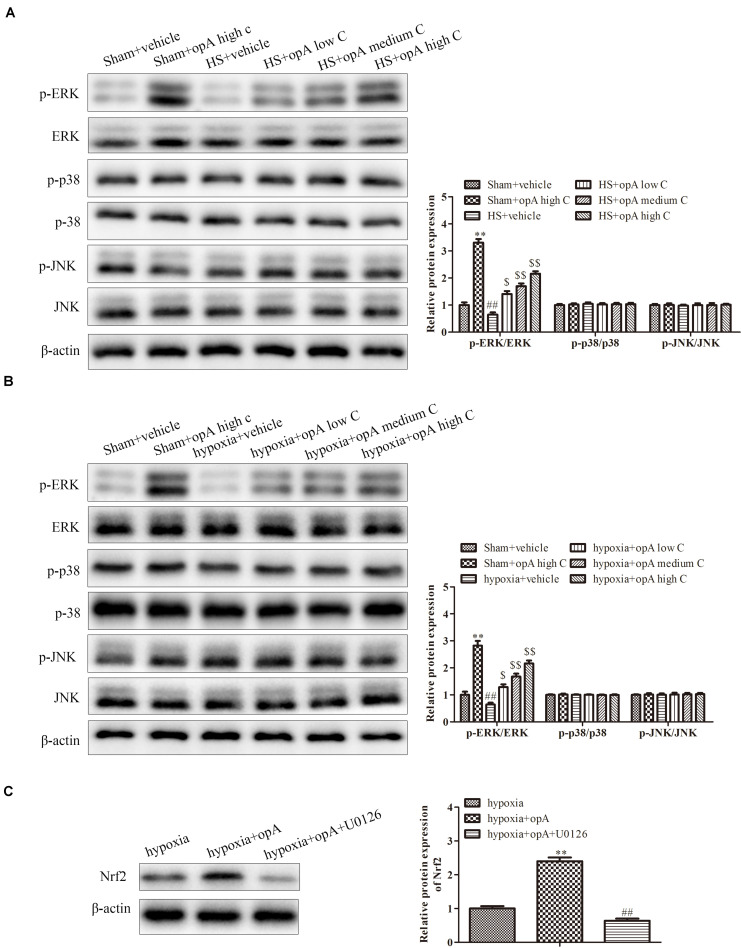
Ophiopogonin A regulates Nrf2 expression by activation ERK signaling pathway. **(A)** Relative protein expression of p-ERK and ERK in sham + vehicle, sham + ophiopogonin A high c, HS + vehicle, HS + ophiopogonin A low c, HS + ophiopogonin A medium c, and HS + ophiopogonin A high c *in vivo*. **(B)** Relative protein expression of p-ERK and ERK in sham + vehicle, sham + ophiopogonin A high c, hypoxia + vehicle, hypoxia + ophiopogonin A low c, hypoxia + ophiopogonin A medium c, and hypoxia + ophiopogonin A high c *in vitro*. **(C)** Relative protein expression of Nrf2 in hypoxia-induced HK-2 cell. Data were presented as mean ± SD. ^∗∗^*P* < 0.01, ^##^*P* < 0.01, ^$^*P* < 0.05, ^$$^*P* < 0.01 compared with the corresponding control group.

Finally, to investigate the related signaling pathway, the expression of p-ERK/ERK, p-p38/p-38, and p-pJNK/JNK was detected by western blot analysis *in vivo* and *in vitro*. The results revealed that ophiopogonin A significantly promoted the phosphorylation of ERK in normal rats (or HS rats) and HK-2 cells (or hypoxia-treated HK-2 cells), especially in high concentration (Figures 6A,B), while the expression of p-p38, p-38, p-pJNK, and JNK had no significant difference. In addition, U0126 (a kind of ERK pathway inhibitor) was used to treat HK-2 cells. We found that U0126 reversed the upregulation of Nrf2 caused by ophiopogonin A in hypoxia-treated HK-2 cells (Figure 6C). These data suggest that ophiopogonin A regulated Nrf2 expression via activating the ERK signaling pathway.

## Discussion

Hemorrhagic shock is a life-threatening disease that leads to systemic inflammatory response syndromes (SIRS) and multiple organ dysfunction syndromes (MODS) (Ulvik et al., 2007; Chen et al., 2013; Sauaia et al., 2017), and is one of the main causes of death among ICU patients (Martel, 2018). Fluid resuscitation is an important method in the treatment of HS, which aims to restore circulating blood volume and give sufficient oxygen to provide tissue metabolism, and enabling endothelial cells to resist ischemia and reperfusion injury (Meizoso et al., 2016; Nguyen et al., 2016). However, the side effects of fluid resuscitation should not be ignored. Rapid replenishment of large amounts of fluid significantly increases inflammatory response, accelerates organ dysfunction, and even death (Semler et al., 2018). The kidney is most vulnerable to HS. Therefore, new strategies and drugs to treat HS need to be explored. In the present study, we observed that the typical symptoms in HS-evoked kidney injury including increased levels of Cr, BUN, KIM-1, NGAL, MPO-positive cells, iNOS, as well as kidney pathological changes and inflammatory damage. While ophiopogonin A, active ingredients extracted from *Ophiopogonis Radix*, improved the renal injury induced by HS. In addition, we had similar results in hypoxia-induced HK-2 cells *in vitro*. Importantly, the protective effect of ophiopogonin A was partly mediated by the Nrf2/p-ERK/ERK signal pathway. Our findings suggest that ophiopogonin A might be a potential therapeutic agent in the treatment of HS-induced kidney injury.

Increasing evidence shows that HS results in the upregulation of various pro-inflammatory factors *in vivo* (Al-Amran et al., 2011). TNF-α, IL-1β, and IL-6 exert critical roles in the development and occurrence of HS-induced renal injury (Faubel et al., 2007). The production of proinflammatory cytokines including TNF-α, IL-1β, and IL-6 also drives the expression of iNOS in various diseases. Good evidence proves that the excessive production of iNOS and inflammatory factors contributes to the occurrence of HS (Cotogni et al., 2010). Studies have shown that ophiopogonin has powerful anti-inflammatory effects. For example, ophiopogonin D alleviates cardiac hypertrophy by inhibiting the activation of the NF-KB pathway and reducing inflammation *in vivo* (Wang et al., 2018). However, the effect of ophiopogonin A has not been demonstrated in the HS. In the study, our results demonstrate that ophiopogonin A significantly suppressed HS-induced inflammatory cytokines TNF-α, IL-1β, and IL-6 production. Moreover, ophiopogonin A was found to inhibit hypoxia-induced TNF-α, IL-1β, and IL-6 production in HK-2 cells. Neutrophils play a key role in the renal injury associated with HS. HS promotes neutrophil infiltration into the tissue, which results in the release of reactive oxygen species (ROS), nitric oxide (NO), and MPO, and causes local cytotoxic effects (Sauaia et al., 2017). We found that ophiopogonin A reduced the increased number of MPO-positive cells in the kidney and hypoxia-induced HK-2 cells, suggesting that ophiopogonin A had protective effects in neutrophil-mediated organ injury.

The mechanism of ophiopogonin A in reducing systemic inflammation and organ damage in HS involves NF-E2-related factor 2 (Nrf2). Part of the Cap‘n’Collar (CNC) transcription factor family (Moi et al., 1994), it plays a key role in maintaining the balance of redox homeostasis by inducing the expression of several genes related to antioxidant defense (He et al., 2020; Lu Y. et al., 2020). Nrf2 deletion or activation disorder leads to cell intolerance or decreased tolerance to oxidative stress, which increases the negative effects of oxidative stress, leading to cell dysfunction, apoptosis, and in some cases, death. Theiss et al. (2009) confirmed that sustained Nrf-2 activation during colitis mediates its inhibition effects against inflammation. Furthermore, some reports indicate the protective effects of Nrf2 in local cerebral ischemia and renal ischemia. Nrf2 eliminates oxygen free radicals in ischemic brain tissue and kidney tissue by increasing glutathione synthesis and that it protects brain cells and kidney cells (Liu et al., 2009; Righy et al., 2016). However, the protective effect of Nrf2 in systemic HS has not been reported. Our study found that ophiopogonin A significantly increased Nrf2 expression in HS mice and hypoxia-induced HK-2 cells. The knockdown of Nrf2 reversed the protective functions of ophiopogonin A *in vivo* and *in vitro*, revealing that ophiopogonin A improved HS-induced kidney injury by the Nrf2 signal pathway.

Extra cellular regulated protein kinase (ERK) is an important member of the MAPK family that responds to a variety of extracellular stimuli and participates in the regulation of proliferation, apoptosis, DNA damage repair, and other cellular life processes (Glaros et al., 2010). The dysfunction of the ERK signal is directly related to the occurrence of neurodegenerative diseases, chronic inflammation, cancer, and many other diseases (Herrero et al., 2015). The ERK pathway is involved in HS-induced a variety of inflammation and tissue damage (Li et al., 2013). This study found that ophiopogonin A promoted the phosphorylation of ERK in HS mice and hypoxia-induced HK-2 cells.

In summary, the results of this study reveal that ophiopogonin A mitigated HS-induced renal damage and inflammatory response. The protective mechanism of ophiopogonin A was through activating the Nrf2/ERK signal pathway. These findings suggest that ophiopogonin A could be an effective drug for the treatment of HS-induced kidney injury.

## Data Availability Statement

The original contributions presented in the study are included in the article/supplementary material, further inquiries can be directed to the corresponding authors.

## Ethics Statement

The animal study was reviewed and approved by the Institutional Animal Care and Use Committee (IACUC) of the Affiliated Hospital of Nantong University.

## Author Contributions

XS conceived and designed the study. YY performed the literature search. JL and JY performed the data extraction. QG and WG completed the experimental operation. FL drafted the manuscript. All authors read and approved the final manuscript.

## Conflict of Interest

The authors declare that the research was conducted in the absence of any commercial or financial relationships that could be construed as a potential conflict of interest.
